# Improved transient electroluminescence technique based on time-correlated single-photon counting technology to evaluate organic mobility

**DOI:** 10.1007/s12200-022-00021-8

**Published:** 2022-04-20

**Authors:** Xianfeng Qiao, Shu Xiao, Peisen Yuan, Dezhi Yang, Dongge Ma

**Affiliations:** 1 Institute of Polymer Optoelectronic Materials and Devices, Guangdong Provincial Key Laboratory of Luminescence from Molecular Aggregates, State Key Laboratory of Luminescent Materials and Devices, South China University of Technology, Guangzhou, 510640 China; 2 Wuhan National Laboratory for Optoelectronics, Huazhong University of Science and Technology, Wuhan, 430074 China

**Keywords:** Mobility, Transient electroluminescence (EL), Time-correlated single-photon counting (TCSPC), Sensitivity, Signal-to-noise ratio (SNR), Device

## Abstract

The transient electroluminescence (EL) technique is widely used to evaluate the carrier mobility in the field of organic light emitting diodes. The traditional analog detection strategy using oscilloscopes is generally limited since the background noise causes an underestimation of the mobility value. In this paper, we utilize time-correlated single-photon counting (TCSPC) to probe the transient EL for mobility calculation. The measurements on tris(8-hydroxyquinoline) aluminum (Alq_3_) show that the electron mobilities obtained using the TCSPC technique are slightly higher than those obtained from the analog method at all the investigated voltages. Moreover, the TCSPC mobilities demonstrate weaker dependence on the root of electrical field compared to the oscilloscope mobilities. These improvements are attributed to the unique principle of TCSPC, which quantifies the EL intensity by counting the number of single-photon pulses, improving its single-photon sensitivity and eliminating the negative impacts of electrical noise. These advantages make TCSPC a powerful technique in the characterization of time-resolved electroluminescence.

**Graphical Abstract:**

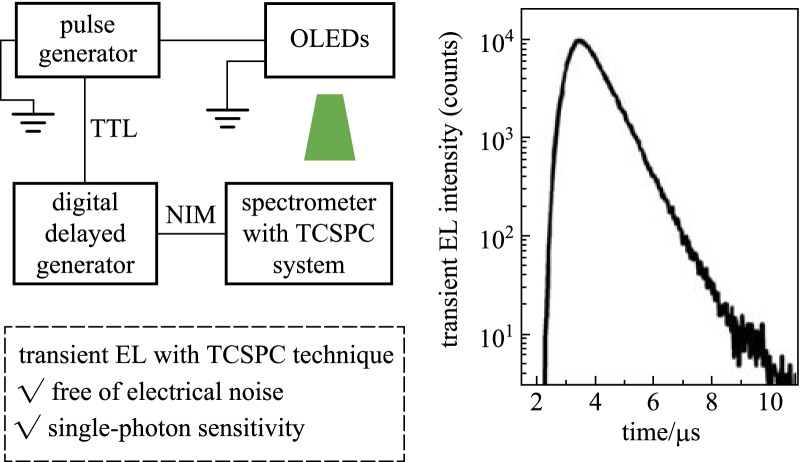

**Supplementary Information:**

The online version contains supplementary material available at 10.1007/s12200-022-00021-8.

## Introduction

Mobility is a fundamental parameter of organic semiconductors that characterizes their transport properties [1], thus evaluating the mobility value is an essential step prior to constructing optoelectronic devices [2–5]. To date, many methods have been developed to measure mobility, including space-charge limited current [6–9], time-of-flight photocurrent [10, 11], transient electroluminescence (EL) [12, 13], dark and light injection transient current [14, 15], impedance spectrum, and transistor techniques [16, 17]. Among these, transient EL is one of the most common techniques used to quantify mobility [18–20].

To implement transient EL, a photomultiplier tube (PMT) and oscilloscope are often employed to probe the transient EL trance, from which the EL turn-on time is calculated. In this process, PMT converts EL into current by an approximate 10^6^ amplification, after which the photocurrent is further transformed into voltage by sampling resistors connected in series. The analog voltage across the resistors is ultimately recorded by a parallel oscilloscope [21]. Essentially, this strategy detects the analog voltage signal, whose amplitude represents the intensity of EL. However, analog detection has two intrinsic weaknesses. First, a random amplification process in PMT will cause a considerable amount of amplitude fluctuation, which contributes noise to the final analog signal. Although this phenomenon can be lessened by taking the average of multiple measurements, the noise cannot be eliminated completely. The second weakness is that the electronic noise of the test system is also amplified and recorded into the results. This noise also prevents the instrument from recording an accurate EL turn-on time. Generally, the turn-on time is overestimated, which causes mobility to be underestimated. Therefore, new strategies with improved photon detection abilities are ideal to precisely and accurately quantify intrinsic mobility.

The time-correlated single-photon counting (TCSPC) technique has been proved as effective for measuring transient EL [22–25]. Instead of detecting the analog signal intensity, the photon counting technique allows for the quantification of single-photon pulses caused by the excitation of dispersed individual photons [26]. Under extremely weak illumination, only one photon could reach the PMT detector and cause a single-photon pulse within the signal period. Then, the arrival time of the corresponding detector pulse after the electrical pulse could be recorded. After an adequate duration of time (signal period), the number of single-photon pulses vs. the arrival time could be measured, which generates the transient EL curve. Essentially, the number of single-photon pulses (not their amplitudes) represents the EL intensity. This unique testing technique is advantageous over analog detection in three ways. Firstly, with optimal settings, the single-photon signal could be individually identified since the amplitude of the background noise signals is much smaller, making TCSPC extremely sensitive when detecting at the single-photon level. Secondly, the electrical noise could be eliminated from the measurement results. Thirdly, TCSPC has a picosecond resolution, which satisfies the demands of transient EL. These unique advantages make TCSPC an ideal alternative to record time-resolved EL for mobility quantification.

**Fig. 1:**
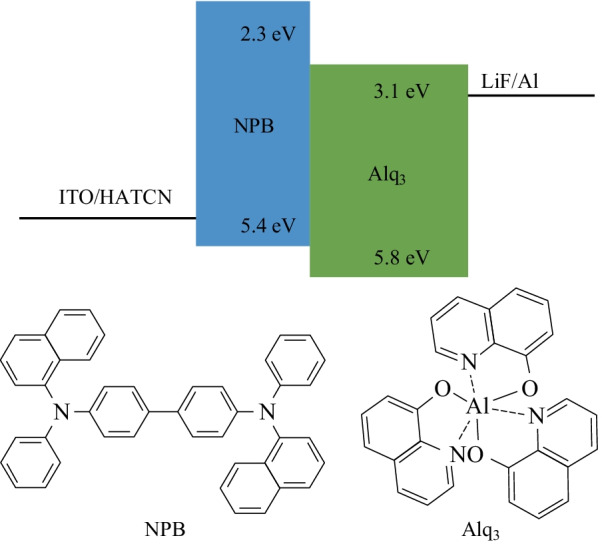
Energy level diagram of device and molecule structures

In this paper, we attempted to utilize the TCSPC technique to investigate and evaluate organic mobility. Analog detection with an oscilloscope was also performed for comparison. TCSPC measurements were carried out on tris(8-hydroxyquinoline) aluminum (Alq_3_), a material widely used in the field of organic light emitting diodes (OLEDs) [27]. The device structure is indium tin oxide (ITO)/1,4,5,8,9,11-hexaazatriphenylene hexacarbonitrile (HATCN, 10 nm)/4,40-Bis[*N*-(1-naphthyl)-*N*-phenylamino]-biphenyl (NPB, 30 nm)/Alq_3_ (60 nm)/LiF/Al. The energy level diagram of the device and molecule structures are also shown in Fig. 1. The resulting TCSPC measurements show shorter turn-on times, thus higher mobility values at all investigated voltages. Meanwhile, the dependence of mobility on the root of electrical field is compared. Finally, the advantages of TCSPC and possible future improvements in the characterization of mobility are further discussed.

## Experimental

### Materials

NPB, Alq_3_, and HATCN were purchased from Jilin OLED Material Tech Co., Ltd. LiF was procured from Aldrich. All materials were used without further purification.

### Device fabrication

The devices were prepared on 180 nm thick, pre-patterned ITO. The ITO substrates were ultrasonically cleaned in diluted detergent for 30 min and washed using deionized water. The substrates were then dried by nitrogen gas and further dried in an oven at 120 °C for 20 min. The organic layer and aluminum cathode layer were grown under vacuum pressure of less than 5.0 × 10^−5^ Pa. The evaporation rate of the organic layer was about 1 Å/s and the evaporation rate of the Al cathode layer was between 3 and 5 Å/s. The emission area of the devices was 4 mm × 4 mm. HATCN and LiF were used as the hole- and electron-injection layers, respectively, to realize the ohmic injection necessary in transient EL measurements. At the same time, 60 nm thick Alq_3_, twice the thickness of NPB, was utilized to ensure that the electron drift time is the limiting factor.

### Device characterization

#### OLED performance

The EL spectra were obtained using a spectrometer (FLS980, Edinburgh Instruments). The density–voltage–luminance characteristics of the current were measured by a source meter (2400, Keithley instruments) and luminance meter (LS110, Konica Minolta). The device performance could be found in the Additional file 1: Figs. S1 and S2.

#### Transient EL measurements

**Fig. 2:**
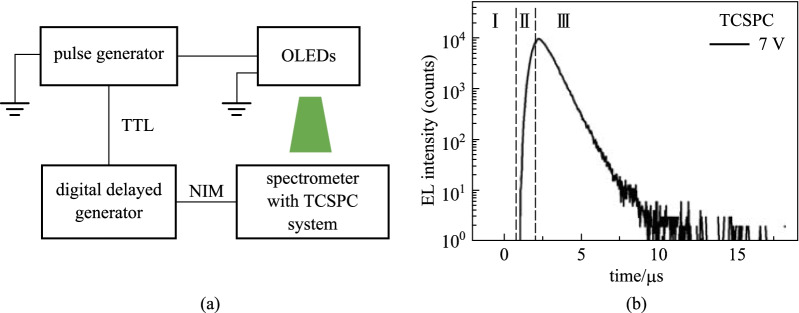
**a** Schematic diagram of the setup with TCSPC technique. **b** Transient EL profile with TCSPC technique at pulse length of 2 µs and amplitude of 7 V

The schematic diagram of the proposed setup is shown in Fig. 2a. In this strategy, a commercial transient spectrometer (FLS980, Edinburgh Instruments) with a proven TCSPC system and a PMT detector (R928P, Hamamatsu Photonics K.K.) is used. The functions of this instrument are similar to the PMT and oscilloscope used in the traditional method. The signal capturing, recording, and processing, as well as date processing, are all carried out by this spectrometer. During this process, the pulse generator (81160A, Keysight) sends the synchronization signal, while the TCSPC module quantifies the number of photons immediately after receiving the trigger signal. Since the synchronization signal of the pulse generator is a TTL signal and the TCSPC module is designed to only receive NIM signals, a digital delayed generator (DG 645, Stanford research system) is used to convert the trigger signal from TTL into NIM form with a 85 ns time delay. The pulse generator works as an internal trigger model, while the digital delayed generator and TCSPC both work as an external trigger model. The generator output voltage has a fixed pulse length of 2 or 5 µs and a frequency of 50 kHz. The pulse amplitude is tunable. The TCSPC module operates at a time range of 20 µs and resolution of about 5 ns. Each measurement autostops at a peak counting of 1000.

For the oscilloscope method, a PMT detector (H11902-110, Hamamatsu Photonics K.K.) and oscilloscope (DSOS104A, Keysight Instruments) are used, with the sampling resistance set at 1 kΩ to balance the detection limit and precision. An average number of 1024 is set for the oscilloscope to smooth the transient EL trace. The pulse generator works as the host to trigger the oscilloscope. The pulse length and frequency are fixed at 10 µs and 1 kHz, respectively.

## Results and discussion

Figure 2b shows a typical transient EL profile measured by TCSPC technique with a pulse length, *t*_P_, of 2 µs and amplitude of 7 V. Three regions are clearly observed in Fig. 2b, which are consistent with the analog detection results [28]. In region I, external pulse is applied and charge carriers are injected from the electrodes. Since the mobility of NPB is approximately two orders of magnitude higher [29], the holes quickly drift across the thinner NPB layer and arrive at the interface. After a drift time of *t*_D_, the electrons with relative lower mobilities begin to meet the hole and recombine into excitons to generate EL from Alq_3_ molecules near the NPB/Alq_3_ interface. In region II, more electrons reach the NPB/Alq_3_ interface and EL continues to increase intensity. Note that the sum of the rising time, *t*_R_, and drift time, *t*_D_, is equal to the pulse width of 2 µs. Here, the drift time of electrons is experimentally extracted by *t*_D_ = *t*_P_ − *t*_R_. In region III, EL begins to decay immediately after the pulse until the excitons are completely consumed. The device is then ready for the next pulse cycle. In this study, we focus on region I, from which the relative lower electron mobility of Alq_3_ is determined by $\mu ={d}^{2}/({t}_{\mathrm{D}}V)$, where *V* is the external pulse amplitude, *d* is the layer thickness, and *t*_D_ is the electron drift time during the EL turn-on.

It is clearly seen from Fig. 2b that the baseline of region I and end of region III is free of noise in the TCSPC spectra. In addition, the EL turn-on point dividing regions I and II is clear. These features are due to the fact that TCSPC records the number, not amplitude, of the single-photon pulses, eliminating background noise from the final results. Prior to EL turn-on, no photon is emitted, thus no signal is detected. A straightforward inference is that the signal-to-noise ratio (SNR) could be still high even for a weak signal [30]. In contrast, analog detection with an oscilloscope gives a noisy background (Fig. 4), since the oscilloscope records both the photon response and electrical noise. The electrical noise level of dark current is determined by the equipment parameter and circuit. In case of a weaker EL intensity that is close to the background noise, the signal is highly affected, as shown in Fig. 4. Using this as comparison, it is safe to conclude that the TCSPC technique is able to quantify the EL turn-on time much more accurately than the traditional analog method.

**Fig. 3:**
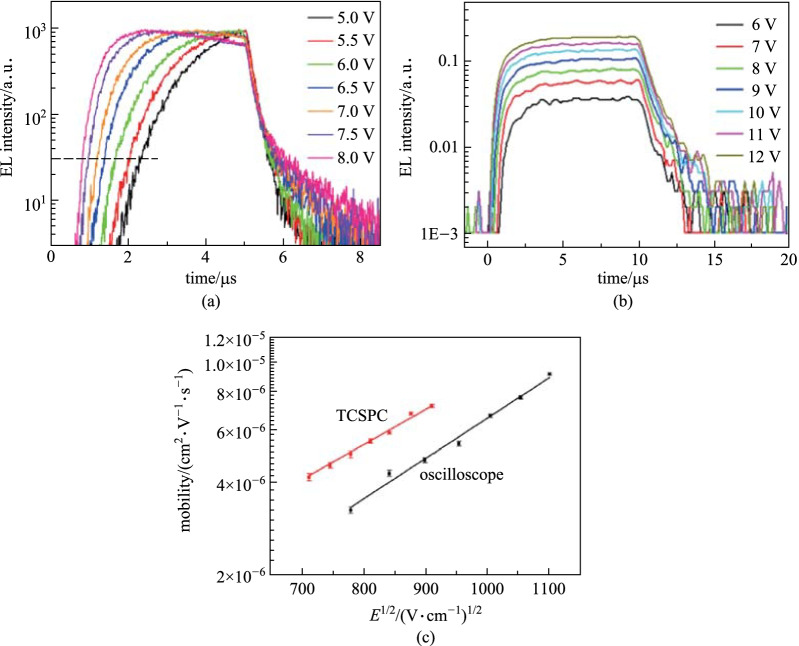
Transient EL profile as a function of voltage for **a** TCSCP and **b** oscilloscope. The dash horizontal line in figure **a** is the virtual baseline due to high background noise. **c** Dependence of mobility on the root of electrical field. The lines are fitting with a linear dependence

Encouraged by the benefits of TCSPC, we also conducted the mobility measurements on real devices. Figure 3a and b show the transient EL curves as a function of pulse amplitude using TCSPC and oscilloscope techniques, respectively. The turn-on point for transient EL is clearly observed in Fig. 3a. Additionally, the values of *t*_D_ were extracted from the transient EL curves to calculate mobility values, which are shown in Fig. 3c, plotted against the root of the electrical field. It can be seen from Fig. 3c that mobility values measured by TCSPC are slightly higher than those measured by oscilloscope. This is because mobility is dependent on ${t}_{\mathrm{D}}^{-1}$ and TCSPC could detect shorter *t*_D_ due to its enhanced photon detection ability. The initial EL intensity is also extremely low at *t*_D_ since only a few electrons have reached the NPB/Alq_3_ interface, thus only TCSPC can detect these signals due to its single-photon sensitivity. Only as time passes and more electrons reach the interface does EL become strong enough for the oscilloscope to detect. Clearly, under the same conditions, the TCSPC technique could detect EL earlier, providing a more accurate *t*_D_ and mobility.

It is also important to note, from Fig. 3c, that both TCSPC and oscilloscope mobilities show a linear dependence on the root of electrical field in the semi-logarithmic plot [1] due to the charge-dipole interaction within the hopping model in the disordered organic semiconductor [31–33]. The slope is $1.16\times {10}^{-3}$ and $1.33\times {10}^{-3}$ for TCSPC and oscilloscope mobilities, respectively. According to the results, oscilloscope mobilities demonstrate stronger dependence, which may also be contributed by the difference in detection ability. As shown in Fig. 3a, the dashed line represents a hypothetical case with higher background noise, where *t*_D_ will be overestimated for all voltages, especially for lower pulse amplitudes. This will eventually cause an overestimation of the mobility dependence.

**Fig. 4:**
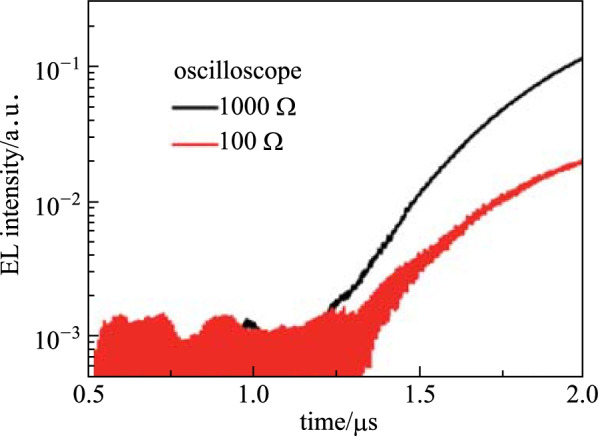
Transient EL profiles measured by oscilloscope with various resistances. The pulse length is 2 µs and amplitude is fixed at 7 V

To provide additional supporting evidence for the deductions above, mobility measurements were taken under different sampling resistances with the same EL illumination. Under these conditions, lower sampling resistances resulted in a decrease in across voltage. In other words, stronger illumination is needed to achieve the same across voltage for lower sampling resistances. In addition, lowering the resistance increased the photon detection limit, which is an opportunity to examine the influence of photon detection ability on mobility quantifying. Figure 4 shows the results under 1 kΩ and 100 Ω resistances using the analog method and the difference is evident at the baseline. As the resistance decreased from 1 kΩ to 100 Ω, the signal-to-noise ratio worsened and the transient EL is smaller at a fixed time. The background noise exhibits a stronger impact with the 100 Ω resistance. Consequently, the *t*_D_ value for 100 Ω is larger than that of 1 kΩ. Taking the above into consideration, it is safe to say that decreasing the photon detection ability causes a greater overestimation of *t*_D_. Again, these findings emphasize the criticality of the photon detection ability for the accurate quantification of mobility.

## Conclusions

In summary, the TCSPC technique has been successfully proved as effective for the quantification of transient EL and organic mobility. When compared to analog detection, TCSPC demonstrates better photon detection limit and sensitivity, as well as elimination of background electrical noise. These advantages are essential to accurately evaluate mobility values and their field-dependence. The experimental results of Alq_3_ using the TCSPC technique show a slightly higher mobility when a constant electrical field is applied and weaker field-dependence at the investigated voltage range. Our results demonstrate that the TCSPC technique is an ideal method in tracing transient electroluminescence and can be further explored in terms of EL device physics.

## Supplementary Information

**Additional file 1: Figure S1.** Device performance. (left) Current efficiency vs current characteristics. (right) Current-voltage-brightness characteristics. **Figure S2.** EL spectrum at various voltages.

## References

[1] Bässler H (1993). Charge transport in disordered organic photoconductors a Monte Carlo simulation study. Phys. Status Solid (b).

[2] Liu J, Zhang H, Dong H, Meng L, Jiang L, Jiang L, Wang Y, Yu J, Sun Y, Hu W, Heeger AJ (2015). High mobility emissive organic semiconductor. Nat. Commun..

[3] O’Neill M, Kelly SM (2011). Ordered materials for organic electronics and photonics. Adv. Mater..

[4] Matsushima T, Bencheikh F, Komino T, Leyden MR, Sandanayaka ASD, Qin C, Adachi C (2019). High performance from extraordinarily thick organic light-emitting diodes. Nature.

[5] Xu K, Hu S, Hu J, Wang X (2019). Numerical simulation of mobility effects on transient electroluminescence spikes in organic light-emitting diodes. J. Electron. Mater..

[6] Mott NF, Gurney RW (1964). Electronic Processes in Ionic Crystals.

[7] Murgatroyd PN (1970). Theory of space-charge-limited current enhanced by Frenkel effect. J. Phys. D Appl. Phys..

[8] Rose A (1955). Space-charge-limited currents in solid. Phys. Rev..

[9] Lampert MA (1956). Simplified theory of space-charge-limited currents in an insulator with taps. Phys. Rev..

[10] Gill WD (1972). Drift mobilities in amorphous charge-transfer complexes of trinitrofluorenone and poly-n-vinylcarbazole. J. Appl. Phys..

[11] Blom PWM, Vissenberg MCJM (1998). Dispersive hole transport in poly(p-phenylene vinylene). Phys. Rev. Lett..

[12] Hosokawa C, Tokailin H, Higashi H, Kusumoto T (1992). Transient behavior of organic thin film electroluminescence. Appl. Phys. Lett..

[13] Braun D, Moses D, Zhang C, Heeger AJ (1992). Nanoseconed transient electroluminescence from polymer light-emitting diodes. Appl. Phys. Lett..

[14] Poplavskyy D, Nelson J (2003). Nondispersive hole transport in amorphous films of methoxy-spirofluorene-arylamine organic compound. J. Appl. Phys..

[15] Knox S, Jones H, Esward T (2010). Device history dependent effects in dark injection transient current measurements of charge mobility in organic light emitting diodes. Proc. SPIE Int. Soc. Opt. Eng..

[16] Lelidis I, Barbero G (2005). Effect of different anionic and cationic mobilities on the impedance spectroscopy measurements. Phys. Lett. A.

[17] Murphy NSJ, Berz F, Flinn I (1969). Carrier mobility in silicon MOST's. Solid-State Electron..

[18] Lee H (2020). Investigation of charge-transport properties in polymer/fullerene blends using transient electroluminescence technique. Jpn. J. Appl. Phys..

[19] Nabha-Barnea S, Gotleyb D, Yonish A, Shikler R (2021). Relating transient electroluminescence lifetime and bulk transit time in OLED during switch-off. J. Mater. Chem. C, Mater. Opt. Electron. Devices.

[20] Shen Q, Hao Y, Ma L, Wang X (2021). Comparative study of red/green/blue quantum-dot light-emitting diodes by time-resolved transient electroluminescence. J. Phys. Chem. Lett..

[21] Xu M, Peng Q, Zou W, Gu L, Xu L, Cheng L, He Y, Yang M, Wang N, Huang W, Wang J (2019). A transient-electroluminescence study on perovskite light-emitting diodes. Appl. Phys. Lett..

[22] Amin NRA, Kesavan KK, Biring S, Lee CC, Yeh TH, Ko TY, Liu SW, Wong KT (2020). A comparative study via photophysical and electrical characterizations on interfacial and bulk exciplex-forming systems for efficient organic light-emitting diodes. ACS Appl. Electron. Mater..

[23] Chen Y, Sun Q, Dai Y, Yang D, Qiao X, Ma D (2019). EL properties and exciton dynamics of high-performance doping-free hybrid WOLEDs based on 4P-NPD/Bepp_2_ heterojunction as blue emitter. Adv. Opt. Mater..

[24] Lin C, Han P, Xiao S, Qu F, Yao J, Qiao X, Yang D, Dai Y, Sun Q, Hu D, Qin A, Ma Y, Tang B, Ma D (2021). Efficiency breakthrough of fluorescence OLEDs by the strategic management of “hot excitons” at highly-lying excitation triplet energy levels. Adv. Funct. Mater..

[25] Xiao S, Qiao X, Lin C, Chen L, Guo R, Lu P, Wang L, Ma D (2022). In-situ quantifying the physical parameters determining the efficiency of OLEDs relying on triplet-triplet annihilation up-conversion. Adv. Opt. Mater..

[26] Beker W, The BH (2017). TCSPC handbook.

[27] Tang CW, Vanslyke SA (1987). Organic electroluminescent diodes. Appl. Phys. Lett..

[28] Kang J, Son JB, Kim GW, Bae S, Min KS, Sul S, Jeon WS, Jang J, Park GS, Shin JK, Kwon JH, Kim SK (2020). Time-resolved electroluminescence study for the effect of charge traps on the luminescence properties of organic light-emitting diodes. Phys. Status Solidi A-Appl. Mater..

[29] Bae HW, Kim GW, Lampande R, Park JH, Ko IJ, Yu HJ, Lee CY, Kwon JH (2019). Efficiency enhancement in fluorescent deep-blue OLEDs by boosting singlet exciton generation through triplet fusion and charge recombination rate. Org. Electron..

[30] Yao J, Ying S, Sun Q, Dai Y, Qiao X, Yang D, Chen J, Ma D (2019). High efficiency blue/green/yellow/red fluorescent organic light-emitting diodes sensitized by phosphors: general design rules and electroluminescence performance analysis. J. Mater. Chem. C, Mater. Opt. Electron. Devices.

[31] Murgatroyd P (1970). Theory of space-charge limited current enhanced by Frenkel effect. J. Phys. D Appl. Phys..

[32] Dunlap DH, Parris PE, Kenkre VM (1996). Charge-dipole model for the universal field dependence of mobilities in molecularly doped polymers. Phys. Rev. Lett..

[33] Parris P, Dunlap D, Kenkre V (2000). Energetic disorder, spatial correlations, and the high-field mobility of injected charge carriers in organic solids. Phys. Status Solidi (b).

