# Supplementary material for: Improved transient electroluminescence technique based on time-correlated single-photon counting technology to evaluate organic mobility
Source: Front Optoelectron. 2022 Apr 20;15(1):11. doi: 10.1007/s12200-022-00021-8 (PMC9756201; doi:10.1007/s12200-022-00021-8)
Supplement: Supplementary file 1 — Additional file 1: Figure S1. Device performance. (left) Current efficiency vs current characteristics. (right) Current-voltage-brightness characteristics. Figure S2. EL spectrum at various voltages. [file 12200_2022_21_MOESM1_ESM.docx]

**Supporting information**

Improved transient electroluminescence technique based on time-correlated single-photon counting technology for evaluating organic mobility

Xianfeng Qiao*, Shu Xiao, Peisen Yuan, Dezhi Yang, Dongge Ma*







Figure 1S device performance, left, current efficiency vs current characteristics. Right, Current-voltage-Brightness characteristics.





Figure 2S EL spectrum at various voltages.
